# Male-selective effects of oxytocin agonism on alcohol intake: behavioral assessment in socially housed prairie voles and involvement of RAGE

**DOI:** 10.1038/s41386-022-01490-3

**Published:** 2022-11-11

**Authors:** Sheena Potretzke, Yangmiao Zhang, Ju Li, Kristopher M. Fecteau, David W. Erikson, Marcel Hibert, Andrey E. Ryabinin

**Affiliations:** 1 Department of Behavioral Neuroscience, School of Medicine, Oregon Health & Science University, Portland, OR 97239 USA; 2 Endocrine Technologies Core, Oregon National Primate Research Center, Oregon Health & Science University, 505 NW 185th Avenue, Beaverton, OR 97006 USA; 3 Laboratoire d’Innovation Thérapeutique, Faculté de Pharmacie, UMR7200 CNRS/Université de Strasbourg, 74 Route du Rhin, F-67412 Illkirch, France

**Keywords:** Addiction, Addiction

## Abstract

Targeting the oxytocin (OXT) peptide system has emerged as a promising new approach for the treatment of alcohol use disorder (AUD). However, further advancements in this development depend on properly modeling various complex social aspects of AUD and its treatment. Here we examined behavioral and molecular underpinnings of OXT receptor (OXTR) agonism in prairie voles, a rodent species with demonstrated translational validity for neurobiological mechanisms regulating social affiliations. To further improve translational validity of these studies, we examined effects of intranasal (IN) OXT administration in male and female prairie voles socially housed in the presence of untreated cagemates. IN OXT selectively inhibited alcohol drinking in male, but not female, animals. Further, we confirmed that exogenously administered OXT penetrates the prairie vole brain and showed that Receptor for Advanced Glycation End-products assists this penetration after IN, but not intraperitoneal (IP), OXT administration. Finally, we demonstrated that IP administration of LIT-001, a small-molecule OXTR agonist, inhibits alcohol intake in male, but not female, prairie voles socially housed in the presence of untreated cagemates. Taken together, results of this study support the promise of selectively targeting OXTR for individualized treatment of AUD.

## Introduction

The prevalence and negative impacts of alcohol use disorder (AUD) demand efficacious treatment options. Currently approved AUD pharmacotherapies show limited success clinically [1–4], despite demonstrated efficacy in preclinical models [5–10]. Oxytocin (OXT), a hormone with essential roles in social behaviors [11, 12], has drawn interest as a potential pharmacotherapy for AUD. Previous research indicated a role for OXT in mediating the processes associated with alcohol use [13]. In particular, OXT’s social effects [14, 15] may serve to bolster abstinence through social support—a key mediator of treatment outcomes [16, 17]. This idea is in line with the previously proposed overarching role of OXT in the regulation of social-cue saliency [18].

Optimization of animal models for testing pharmacotherapies for AUD may help to bridge the gap between preclinical and clinical efficacy. In the current studies, we administered OXT intranasally (IN)—a translationally relevant route exclusively used in clinical trials in human patients. Among the obstacles in development of efficacious therapies for AUD that current therapies have inadequately addressed are the complex interactions of alcohol-related behaviors with the social environment [19–21]. Here we examined the effect of pharmacologically targeting the OXT system in socially housed prairie voles given unrestricted access to the other animals in the cage and alcohol in a continuous-access two-bottle choice paradigm (CA-2BC). Prairie voles are facultatively monogamous rodents with demonstrated translational validity for mechanisms regulating social behaviors, in which the OXT system has been extensively explored [22–24]. As animals that voluntarily consume alcohol, and show social influences on alcohol consumption similar to humans, prairie voles serve as an ideal animal model for these studies [25, 26]. Finally, we used a mixed-cage design, with treated and control animals in the same cage. This design better recapitulates scenarios human patients experience during medication-assisted maintenance of abstinence from alcohol.

Despite previous demonstrations of OXT’s efficacy in decreasing measures associated with AUD preclinically [27–32] and clinically [33–35], the molecular mechanisms of OXT’s effects remain elusive including its transport into the brain. OXT is thought to cross the blood-brain barrier (BBB) [36]. Evidence indicates that OXT acts centrally to inhibit alcohol intake. In rats, centrally administered OXT decreased reinstatement of alcohol self-administration, and central, but not peripheral, administration of a BBB non-penetrant oxytocin receptor (OXTR) antagonist prevented OXT’s inhibitory effects on alcohol self-administration [31, 33]. In mice, a centrally- but not peripherally acting- OXTR antagonist prevented a decrease in alcohol intake following chemogenetic activation of hypothalamic OXT neurons [37]. Recent studies in rodents and non-human primates (NHPs) showed small proportions of exogenous OXT in the brain when administered peripherally, and minute amounts in various brain regions when administered IN [38–41]. However, the precise mechanisms of OXT’s transport into the brain remain unclear [42, 43]. Recent demonstration of OXT’s transport into the brain via the Receptor for Advanced Glycation End-products (RAGE) [44, 45] suggested this protein as a possible mediator of transport. Despite substantial research on RAGE signaling in humans [46–48] and mice [45, 49], the existence of RAGE has not been previously demonstrated in prairie voles. Therefore, we tested whether brain penetrance of exogenous OXT could be facilitated via RAGE by first evaluating its expression in the brain of prairie voles, and then assessing brain levels of exogenously administered stable isotope-labeled OXT in the presence or absence of a RAGE antagonist, using liquid chromatography-tandem triple quadrupole mass spectrometry (LC-MS/MS) for quantification.

Determining the behavioral and molecular effects of OXT on alcohol consumption is important for translational application and optimization of treatment efficacy. However, OXT has poor pharmacokinetic properties: it is not orally available (limiting the administration to IN, IP, or intravenous routes), displays low bioavailability, and is metabolically unstable [50, 51]. Given OXT’s low brain-penetrance and potentially non-specific effects on the related vasopressin receptor AVPR1a, development of an OXTR receptor agonist with a more desirable pharmacokinetic profile could increase the translational potential of targeting the OXT system [52, 53]. While three small-molecule OXTR agonists have been reported [52–55], two (TC-OT-39 and WAY-267646) have moderate affinity for the AVPR1a [54, 56]. The most recently developed small-molecule OXTR agonist, LIT-001, not only has a high affinity for the OXTR, but also has a several magnitudes lower affinity to AVRP1a. LIT-001 was effective in restoring social interaction in a mu opioid receptor knockout animal model of Autism Spectrum Disorder [54], as well as in inhibiting hyperalgesia caused by inflammation [57]. To assess efficacy of LIT-001 in decreasing alcohol consumption similarly to IN OXT in socially housed prairie voles, we utilized a CA-2BC paradigm and mixed-cage treatment design.

Taken together, our experiments investigated effects of IN administration of OXT on voluntary alcohol intake in socially housed prairie voles, the contribution of RAGE to the brain penetrance of OXT, and the ability of an OXTR agonist to modulate alcohol intake in this alcohol drinking model. With consideration of the known sex differences in OXT functioning [11], all experiments were performed with both male and female animals.

## Materials and methods

### Animals

Adult female and male prairie voles from our laboratory’s colony at Oregon Health & Science University (OHSU) were assessed in these experiments (see Supplementary Material). All experiments were approved by the Institutional Animal Care and Use Committee at OHSU, Portland, OR, USA and conducted in accordance with the National Institutes of Health (NIH) Guidelines for the Care and Use of Laboratory Animals.

### Experimental design

For experiments examining the effects of pharmacologically targeting the OXT system on alcohol (and water) consumption, adult female and male prairie voles were given *ad libitum* access to alcohol in a CA-2BC design for 6 days. Previous studies indicated that withdrawal from an equivalent voluntary alcohol exposure resulted in hyperalgesia, suggesting that this exposure is a model of at least mild alcohol dependence [58]. Fluid consumption was measured before and after treatment (IN OXT or IP LIT-001, or vehicle) using the Herdsman-2 (HM2; MBrose Faaborg, Denmark) cage system. The HM2 system is designed to allow socially housed animals unrestrained access to and interaction with other animals in the cage, while accounting for precise, individualized measures of fluid intake through the combined use of balances and radiofrequency identification (RFID) implants. This cage system has been previously utilized to assess potential pharmacotherapies for AUD in rodents [59, 60]. Further details are provided in the Supplementary Material (Supplementary Fig. S1). Consistent with previous studies [27, 60], a mixed-cage design (treated and control animals in the same cage) was used.

Following confirmation of the presence of RAGE mRNA and protein in prairie vole brain using RT-PCR and immunohistochemistry as described in the Supplementary Material, we tested whether IN or IP administered OXT penetrates into the brain and whether the RAGE protein is involved in this transport. Adult female and male prairie voles were assigned to control or treatment groups. Animals in the treatment group received the high affinity, BBB-permeant RAGE antagonist FPS ZM1 and control animals received saline 30 min prior to administration of oxytocin-(*leucine*-5,5,5-d_3_, *glycine*-2,2-d_2_) trifluoroacetate salt (d5 OXT) in saline to all animals. After 10 min, animals were deeply anaesthetized under 4% isoflurane and perfused with heparinized saline. This time period was based on evidence in mice and rats showing that peripheral OXT treatment rapidly increased brain microdialysate and plasma OXT levels during the first 30 min following treatment [61]. Brains were extracted and snap-frozen in isopentane, then stored at −80 °C until prepared for analysis via LC-MS/MS (Supplementary Fig. S2).

### Drugs

OXT acetate salt (Bachem, Torrence, CA, USA) was dissolved in normal saline to doses of 5.0 mg/kg and 10.0 mg/kg (25 µl, IN). LIT-001 was synthesized in the laboratory of Dr. Marcel Hibert. It was dissolved in 5% dimethyl sulfoxide in saline and administered at a dose of 10 mg/kg (IP). FPS ZM1 (1 mg/kg, IP; Tocris, Minneapolis, MN, USA) and d5 OXT (3 µg/25 µl, IN or 12 µg/0.1 ml, IP; Sigma-Aldrich, St. Louis, MO, USA) were dissolved in saline. The d5 OXT dose was based on previous assessments of OXT penetrance [39].

### Experimental measures and statistical analysis

Alcohol and water intake were calculated for each animal as liquid consumed in grams (adjusted per density of 5% (v/v) ethanol and water) over body weight in kilograms. Alcohol and water drink size were determined by the volume of liquid consumed (ml) per consumption event. Consumptive visits were defined as channel entries during which a consumption event was recorded. Non-nutritive visits were defined as channel entries which did not result in a consumption event. Cumulative measures were analyzed at hourly intervals for 24 h post-treatment. Concentrations of d5 OXT (pg/mg of tissue) were obtained through LC-MS/MS analysis on a Shimadzu Nexera-LCMS-8060 instrument using a previously established method [39]. Data analysis was performed in IBM SPSS Statistics 27 and visualization was performed in Prism 9.3.1. The distribution of many of these measures was not normal. Therefore, non-parametric statistics were applied, including Kruskal–Wallis test and post hoc analyses with Mann–Whitney *U*-test. While Results below only include *p* values, detailed statistical analyses are presented in Supplementary Material Table S1.

## Results

### Effects of IN OXT on alcohol consumption

**Fig. 1:**
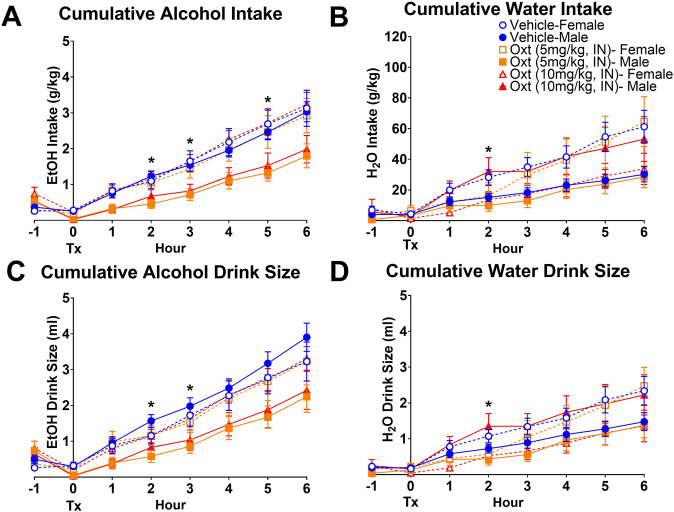
Cumulative measures of alcohol and water consumption from 1 h pre- and through 6 h post-intranasal oxytocin treatment. Significant differences were observed between groups in alcohol intake at 2, 3 and 5 h post-treatment, while differences approached significance at 1 and 4 h (**A**). Significant differences were also observed in alcohol drink size at the 2 and 3 h time points (**C**). OXT also affected water intake (**B**) and drink size (**D**) at 2 h. Data are presented as mean ± standard error of mean. *p < 0.05, Kruskal–Wallis test. Tx denotes time of treatment. Note: −1 h time point is included to demonstrate lack of differences between groups prior to treatment and is defined as the cumulative measure during the 1 h prior to treatment. Cumulative measures post-treatment begin at the 0 h time point which encompasses the time of treatment (Tx) through the first hour. *N:* female vehicle = 29, female 5 mg/kg OXT = 13, female 10 mg/kg OXT = 14, male vehicle = 27, male 5 mg/kg OXT = 12, male 10 mg/kg OXT = 16.

Cumulative measures of intake and associated behaviors were examined at hourly intervals (Fig. 1A). There were no pre-treatment differences in alcohol or water intake between animals assigned to the groups. This was confirmed at both 1 h prior to treatment (−1 h, *p*s > 0.18) and at the time of treatment (0 h, *p*s > 0.38). Differences in alcohol intake approached significance at the 1 h post-treatment time point (*p* = 0.08) and were found to be non-specific to alcohol at 2 h post-treatment—both doses of OXT decreased alcohol intake, but 10 mg/kg of OXT increased water intake in males (alcohol: *p* = 0.02; water: *p* = 0.04, Fig. 1B). However, at 3 h post-treatment, alcohol intake was significantly different between groups (*p* = 0.02), without differences in water intake (*p* = 0.11). A similar pattern of effects was observed for drink size (Fig. 1C, D). While significant differences between groups in drink size at the 2 h time point were observed for both alcohol (*p* = 0.02) and water (*p* = 0.03), these differences were alcohol-specific at 3 h time point (*p* = 0.04). Differences in alcohol intake approached significance at 4 h (*p* = 0.054) and were significant at 5 h (*p* = 0.03). No differences in alcohol drink sizes were found at these time points (*p*s > 0.07). No significant differences were observed in alcohol or water consumptive visits (Supplementary Fig. S3A, B) or non-nutritive visits (Supplementary Fig. S3C, D) at any time point (*p* > 0.94).

**Fig. 2:**
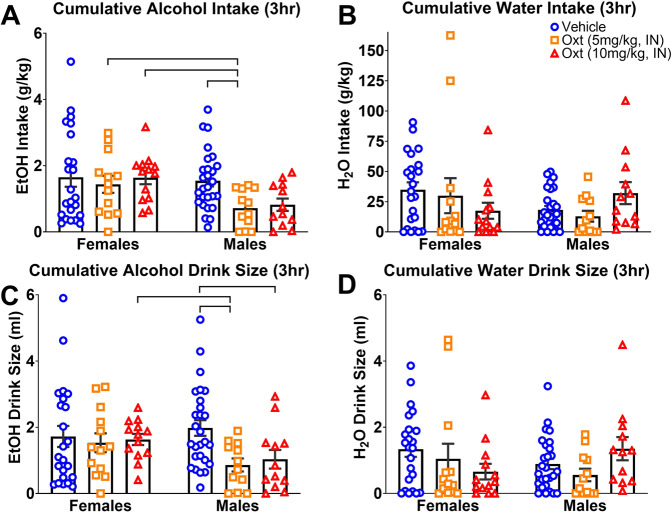
Cumulative measures of alcohol and water consumption 3 h following intranasal oxytocin treatment. OXT was observed to selectively decrease measures of alcohol consumption 3 h post-treatment; thus, group comparisons were confirmed. Male prairie voles treated with 5 mg/kg and 10 mg/kg IN OXT consumed significantly less alcohol and displayed smaller drink sizes than their saline control counterparts (**A**, **C**). No effect was observed on water intake or drink size (**B**, **D**). Data are presented as mean ± standard error of mean. Brackets indicate statistically significant differences between groups at *p*s < 0.05, Mann–Whitney test. *N* values are the same as in Fig. 1.

Post hoc analysis at the 3 h post-treatment interval showed that male prairie voles treated with 5 mg/kg and 10 mg/kg IN OXT displayed lower alcohol intake (*p* = 0.01 and *p* = 0.02, respectively) and smaller drink sizes (*p* = 0.01 and *p* = 0.02, respectively) than their control counterparts (Fig. 2A, C). Males treated with 5 mg/kg IN OXT displayed lower levels of alcohol intake than both 5 mg/kg (*p* = 0.04) and 10 mg/kg (*p* = 0.01) OXT-treated females, as well as smaller drink sizes than 10 mg/kg OXT-treated females (*p* = 0.01). Males treated with 10 mg/kg IN OXT also displayed lower levels of alcohol intake (*p* = 0.01) and drink size (*p* = 0.06) than their female 10 mg/kg IN OXT counterparts. No significant differences were observed in any other measure of consumption (Fig. 2B, D and Supplementary Fig. S4).

Taken together, these data identify a sex-dependent effect of IN OXT on alcohol consumption. OXT significantly decreased alcohol intake in males, without affecting alcohol consumption in females. Analysis of drinking patterns suggested that OXT’s effects were primarily due to the drink size and less due to the number of consumptive or non-consumptive visits to the alcohol spout.

### Brain levels of exogenously administered d5 OXT and involvement of RAGE in its transport

**Fig. 3:**
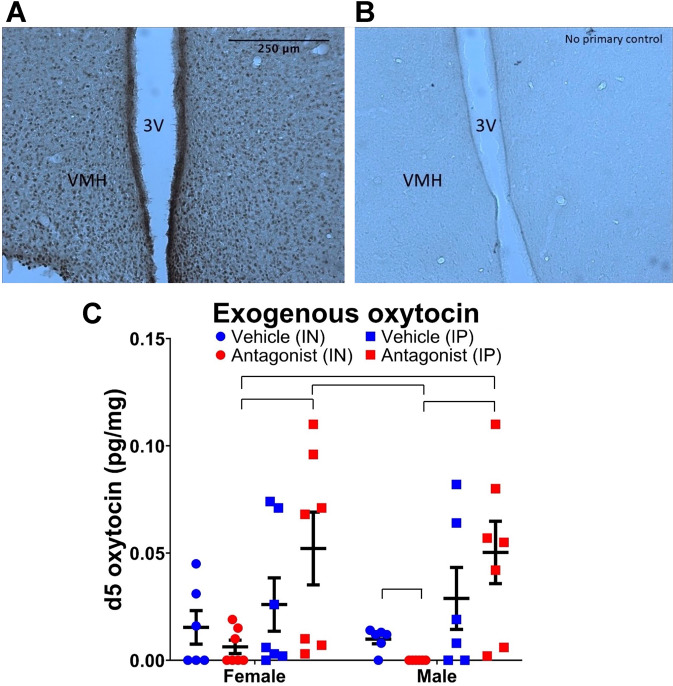
RAGE expression in hypothalamus and brain d5 OXT levels following exogenous administration. RAGE immunoreactivity was observed widely in the prairie vole brain, including areas surrounding the ventricles (3V) and ventromedial hypothalamus (VMH) (**A**). Specificity of staining was confirmed by control immunohistochemical reactions omitting primary antibodies (**B**). Images **A** and **B** were taken at ×20 objective magnification. LC-MS/MS analysis detected d5 OXT in the brain (**C**). Pre-treatment with the RAGE antagonist FPS ZM1 significantly decreased exogenous d5 OXT levels compared to vehicle pre-treated controls in males when administered intranasally. Pre-treatment with antagonist also resulted in decreased d5 OXT levels in females and males when administered IN vs. IP. Data are presented as mean ± standard error of mean. Brackets indicate statistically significant differences between groups at *p*s < 0.05, Mann–Whitney test. *N*: female vehicle (IN) = 6, female antagonist (IN) = 7, female vehicle (IP) = 7, female antagonist (IP) = 7, male vehicle (IN) = 6, male antagonist (IN) = 6, male vehicle (IP) = 6, male antagonist (IP) = 7.

To test whether exogenously administered OXT reaches the brain in prairie voles, we examined levels of deuterium-labeled OXT (d5 OXT) with LC-MS/MS after IN and IP administration. In addition, since RAGE can act to transport OXT into the brain in mice, we tested whether it can also help transport OXT to the brain in voles [44, 45]. To this end, we first confirmed RAGE expression in prairie vole brain using RT-PCR and immunohistochemistry. We found that RAGE was widely expressed in the brain, including the hypothalamus (Fig. 3A, B and Supplementary Fig. S5). Using LC-MS/MS, we detected d5 OXT in brains of male and female prairie voles (Fig. 3C). Significant differences in d5 OXT levels were observed between groups (*p* = 0.01). Post-hoc analysis showed significantly lower levels of d5 OXT in RAGE antagonist pre-treated males vs. vehicle controls following IN administration (*p* = 0.02). Significantly lower d5 OXT levels were also observed in antagonist pre-treated females that received IN d5 OXT compared to IP d5 OXT (*p* = 0.04), as well as to antagonist pre-treated males that received IP d5 OXT (*p* = 0.02). Similarly, significantly lower d5 OXT levels were observed in antagonist pre-treated males that received IN d5 OXT compared to IP d5 OXT (*p* = 0.001), as well as to antagonist pre-treated females that received IP d5 OXT (*p* = 0.001). No significant differences were observed between control and antagonist pre-treated female or male animals when d5 OXT was administered IP (*p*s > 0.21), and no significant differences were observed between control animals according to route of d5 OXT administration (*p*s > 0.45).

These results indicate that exogenously administered OXT, specifically via the translationally relevant IN route, penetrated the prairie vole brain in a process at least partially dependent on RAGE-mediated transport.

### Effects of LIT-001 on alcohol consumption

**Fig. 4:**
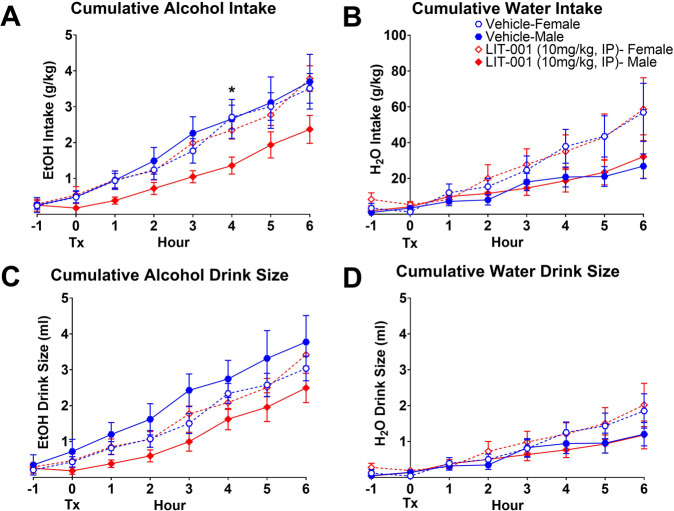
Cumulative measures of alcohol and water consumption from 1 h pre- through 6 h post-LIT-001 treatment. Significant differences were observed between groups in alcohol intake at 4 h post-treatment (**A**), without any effect on water intake (**B**). No differences were observed in alcohol (**C**) or water (**D**) drink size. Data are presented as mean ± standard error of mean. *p < 0.05, Kruskal–Wallis test. Tx denotes time of treatment. Note: −1 h time point included to demonstrate lack of differences between groups prior to treatment and is defined as the cumulative measure during the 1 h prior to treatment. Cumulative measures post-treatment begin at the 0 h time point which encompasses the time of treatment (Tx) through the first hour. *N*: female vehicle = 15, female LIT-001 = 16, male vehicle = 12, male LIT-001 = 11.

To test whether an OXTR agonist with a non-peptide structure and more specific affinity for OXTR could be used in place of OXT, we examined the effects of LIT-001 on cumulative measures of alcohol and water consumption. Cumulative measures of intake and associated behaviors were examined at hourly intervals. LIT-001 decreased cumulative alcohol intake (*p* = 0.05) at 4 h post-treatment (Fig. 4A), without affecting water intake (*p* = 0.46; Fig. 4B). Treatment with LIT-001 did not significantly affect alcohol or water drink size (*p*s > 0.14; Fig. 4C, D) or number of alcohol and water non-nutritive visits (*p*s > 0.58; Supplementary Fig. S6C, D). However, group differences in alcohol consumptive visits were noted at the 4 h post-treatment time point (*p* = 0.03; Supplementary Fig. S6A), without differences in water consumptive visits (*p* = 0.56; Supplementary Fig. S6B).

**Fig. 5:**
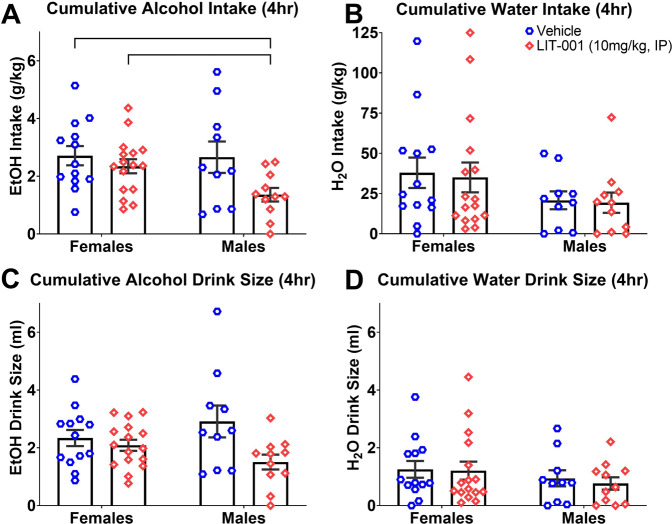
Cumulative measures of alcohol and water consumption 4 h following LIT-001 treatment. Despite observable differences, males treated with LIT-001 did not consume significantly less alcohol than vehicle-treated male controls, but did consume less than both female controls and LIT-001-treated females (**A**), without any differences observed in water consumption (**B**). No differences were observed between groups in alcohol (**C**) or water (**D**) drink size. Data are presented as mean ± standard error of mean. Brackets indicate statistically significant differences between groups at *p*s < 0.05, Mann–Whitney test. *N* values are the same as in Fig. 4.

Post-hoc analysis at the 4 h interval revealed that LIT-001 was selectively effective in decreasing cumulative alcohol intake (*p* = 0.05), as well as consumptive visits to the alcohol channel. The difference in alcohol intake between males treated with LIT-001 and their male vehicle control counterparts did not reach statistical significance (*p* = 0.10); however, significant differences were found between LIT-001-treated males and female controls (*p* = 0.01), as well as their LIT-001-treated female counterparts (*p* = 0.02; Fig. 5A). Treated males also made fewer alcohol channel consumptive visits than both control males (*p* = 0.01) and LIT-001-treated females (*p* = 0.03; Supplementary Fig. S7A). No other significant differences between groups were noted in any of the other measures (Fig. 5B–D and Supplementary Fig. S7A–D).

These data show that LIT-001 selectively decreased cumulative alcohol intake up to 4 h post-treatment in males, without affecting consumption levels in females, which offers promising evidence that a small-molecule OXTR agonist can substitute OXT to affect alcohol consumption.

## Discussion

In total, these studies demonstrate: (1) IN OXT treatment sex-specifically decreases alcohol consumption in a translationally-relevant rodent model in settings which recapitulate scenarios human patients may face, i.e., in the presence of non-treated peers drinking alcohol, (2) RAGE is widely expressed throughout the prairie vole brain and is involved in the transport of OXT into the brain following IN administration, and (3) LIT-001, a small-molecule OXTR agonist can replace OXT with a similar, sex-specific effect in decreasing alcohol consumption.

Both OXT (IN) and LIT-001 (IP) were effective in decreasing cumulative alcohol consumption hours after treatment in males, demonstrating the plausibility of targeting the OXT system as a pharmacotherapy for AUD. None of the inhibitory effects on alcohol intake were due to potential non-specific effects on fluid consumption. In the current study, we did not test these treatments in single-housed animals. Based on demonstrations of effects of IN OXT in single-housed rats [31], we expect that IN OXT and IP LIT-001 would also be effective in decreasing alcohol intake in single-housed prairie voles. While future direct quantitative comparison of these treatments across various housing conditions would be interesting, our findings demonstrate that targeting the OXTR can decrease alcohol intake in the presence of untreated individuals.

Despite the short half-life of estimated tens of minutes [51], OXT continued to be effective for several hours post-administration. This observation suggests a feed-forward effect of OXT, whereby inhibition of the reinforcing effects of alcohol by OXT influences animals’ consummatory behavior after the peptide is eliminated from the system. Since the half-life of LIT-001 is estimated to be longer than 1 h [57], it is interesting that the observed time course of effect of LIT-001 was similar to that of OXT, a finding suggesting that a different dose could be more effective. While a statistically significant difference was not observed at 4 h between LIT-001-treated and control males, alcohol intake levels in treated males were nearly half of those of control males (Table S1). This sizable decrease could signify considerable harm reduction. Moreover, alcohol intake levels in control males were only slightly lower than control females, which were found to be significantly different from LIT-001-treated males. Despite a slight reduction, LIT-001-treated females consumed similarly to female and male saline controls.

These sex-specific effects of targeting the OXT system on alcohol intake have been hypothesized previously [62]. However, this hypothesis was mainly based on differential effects of excessive alcohol on hypothalamic OXT and OXTR levels in male vs. female subjects. In contrast to this observation, alcohol consumption decreases hypothalamic OXT levels in both male and female prairie voles [63, 64]. Similarly, male-specific effects of the OXT activity are unlikely due to differential brain penetrance as indicated by the lack of significant sex differences in d5 OXT levels in our LC/MS-MS experiment. This observation agrees with the lack of sex differences in brain penetration of d5 OXT in NHPs [39]. Nevertheless, these differences in effects could have other molecular explanations. OXT’s central functions are critically dependent on steroid hormones, in particular estrogens [65–69]. However, since female prairie voles are induced ovulators, and female and male prairie voles were housed separately, at least fluctuations in estrogen levels were unlikely to contribute to the lack of significant effects in females. More relevantly, sex differences in OXT and OXTR distribution have been reported in animals, including mice, rats, prairie voles and NHPs [70–73]. OXT and OXTR expression is typically higher in females [70, 74], but areas such as the ventromedial hypothalamus in rats, or medial prefrontal cortex in prairie voles show higher OXTR binding in males vs. females [75, 76]. From a more global perspective, given the importance of OXT for female reproductive and maternal behaviors, social- and drug-targeted behaviors in females could be more resilient to fluctuations in OXT activity.

Interestingly, while the relatively equal levels of alcohol intake between IN OXT- and IP LIT-001-treated females and their respective controls suggest a lack of an effect, it is also possible that this observation is signaling a different type of effect in females. Thus, according to the Social Salience Hypothesis of OXT functioning [18], the salience of social stimuli (i.e., conspecifics) may be augmented over alternative rewards, such as alcohol. Indeed, IN OXT can have different effects on processing of social stimuli in women and men. For example, women have indicated more distress and aggression in response to a social stress test and men have reported fewer negative effects [77]. Given prior demonstrations of social facilitation and inhibition of alcohol consumption in co-housed same-sex animals [25, 78], OXTR agonism may be acting to enhance the saliency of social cues in females and thus result in animals matching consumption with one or more animals in the cage. Future investigations could compare social interactions and consumption patterns between animals to determine whether effects on social salience contribute to differential effects of OXTR agonism on alcohol intake in males vs. females.

We also demonstrate here, for the first time, the presence of RAGE in the prairie vole brain. Importantly, RAGE immunoreactivity was detected in the choroid plexus, hypothalamus and hippocampus, confirming expression in possible locations of central penetration. Together with previous demonstrations of RAGE’s role in the transport of OXT across the BBB [45], our findings support a potential transporter role for RAGE in the blood-CSF barrier, but indicate that this role might vary in different routes of administration. Studies in mice noted that the IN route of administration can result in higher brain bias for the exogenously administered OXT than the IP route [41]. While we did not find significant differences in baseline brain levels of d5 OXT between the two routes of administration, pre-treatment with a RAGE antagonist resulted in a lower level of d5 OXT in the brain after IN, but not IP, administration of OXT. Interestingly, the differential contribution of RAGE to effects of IN vs. IP OXT is in agreement with their varied effects on alcohol intake. For example, IN OXT had more specific inhibitory effects than IP OXT on alcohol drinking in a rat model of severe alcohol dependence [31]. In agreement with greater specificity of this route, IN OXT decreased alcohol intake, but not water intake, in male, but not female, voles in the current study; whereas IP OXT decreased alcohol and water intake in both sexes in a previous prairie vole study [32]. Taken together, this evidence suggests that clinical studies, which predominately use the IN route, will benefit from taking advantage of the potential importance of RAGE in the studied effects of OXT. On the other hand, these observations also indicate that rodent IP OXT studies could have limited translational validity not only because they use a route that is not used in clinical studies, but also because the IP route does not engage OXT transport via RAGE-- which occurs in the clinically relevant IN route. The ability of LIT-001 to selectively decrease alcohol intake following IP administration suggests that small-molecule OXTR agonists could be developed allowing to avoid the inconvenience of IN delivery of OXT in clinical settings. In addition, future studies use of small-molecule OXTR agonists for therapeutic purposes via other routes could overcome the dependence on RAGE as a transporter of OXT.

These results offer further support for the potential of OXT as a pharmacotherapy for AUD. However, many questions remain requiring further examination in future studies. OXT functioning is complex, involving a number of brain areas and systems with roles in processing stimuli, and can affect various physiological and behavioral responses. It is possible that OXT acts through several related systems to exert its effect on alcohol consumption (e.g., altering reward/motivation via dopaminergic mesolimbic systems, moderating effects of stress and promoting allostasis via the HPA axis) and thus could be effective in treating various aspects of AUD (e.g., alcohol tolerance, craving, withdrawal, relapse, etc.). Given OXT’s anxiolytic properties [79, 80] and the reported potential to decrease withdrawal [35], whether its effects on alcohol consumption are mediated by decreases in anxiety-like and withdrawal-like behaviors could be examined. Importantly, given OXT’s known social effects, future studies could measure affiliative behaviors in tested animals to assess whether the decrease in alcohol consumption is mediated by increasing the salience of social reward over the alcohol reward [11–13]. This would be an insightful research direction as the OXT system possesses the distinct potential to harness its social effects to not only offer an alternative source of (social) reward, but also to bolster abstinence through increased social support—a key mediator of treatment outcomes [16, 17].

## Supplementary information

Supplemental Material

Supplemental Table
