# Supplementary material for: Male-selective effects of oxytocin agonism on alcohol intake: behavioral assessment in socially housed prairie voles and involvement of RAGE
Source: Neuropsychopharmacology. 2022 Nov 11;48(6):920–8. doi: 10.1038/s41386-022-01490-3 (PMC10156683; doi:10.1038/s41386-022-01490-3)
Supplement: Supplementary file 1 — Supplemental Material [file 41386_2022_1490_MOESM1_ESM.docx]

**S. Potretzke, Y. Zhang, Ju Li, K.M. Fecteau, D.W. Erikson, M. Hibert, A.E. Ryabinin, Male-selective effects of oxytocin agonism on alcohol intake: Behavioral assessment in socially housed prairie voles and involvement of RAGE**

# **Supplemental Material**

# **Detailed Methods**

## *Housing conditions*

Animals were weaned at age 21 days (±1 day) and housed in same-sex groups (typically siblings, maximum 4 animals) in standard cages (27 x 27 x 13cm) with (1/8in) pelleted cellulose bedding (Biofresh^™^, Patterson, NY, USA), cotton nestlets and Enviro-Dri for nest building/thermoregulation, and wood blocks and Manzanita sticks for chewing/enrichment. It should be noted that female prairie voles are induced ovulators and therefore do not display estrous cycles unless directly exposed to / housed with a male. Female and male animals were housed in separate rooms prior to experiments and separate cages throughout; preventing induction of ovulation and negating need for assessment or tracking of estrous stages. *Ad libitum* access to water and a diet of rabbit chow (Laboratory Rabbit Diet High Fiber, 5326; LabDiet^®^, St. Louis, MO, USA) and Timothy hay (Hand-Selected Timothy Grass; Standlee Premium Products, Kimberly, ID, USA) was provided throughout the course of experiments. Animals experienced a 14:10 light : dark cycle, with lights-on at 06:00.

## *Apparatus*

The Herdsman-2 (HM2) system was used in the experiments measuring alcohol consumption. The HM2 cage system consists of Techniplast 1500U Eurostandard type IV S (48 x 37.5 x 21cm) rodent housing cages equipped with two (11cm) channels (front and back) on one side and stairs specially designed to accommodate prairie voles. Each channel leads to a bottle (one water, one 5% ethanol in water, v/v) below which is a precision balance with a catch tray for spillage- which is automatically deducted from consumption calculations. Breakage of the photobeam at the entrance of the channel triggers the RFID reader to register the animal. A consumption event is initiated when the animal touches the drinking spout and a change of weight of at least 0.02 g is detected; the starting weight is saved and ending weight is registered only after the system receives a stable reading for 5 s. A consumption event is ended 30s after the last touch of the drinking spout, regardless of whether the animal remains in the channel. The system collects a number of measures, including: weight of liquid consumed, number of channel entries and consumptive vs. non-nutritive visits (whether liquid is consumed or not). Bedding, nesting/thermoregulating materials and enrichment conditions in HM2 cages were identical to the home cage, with the addition of a 10in Habitrail OVO tube (Habitrail, Hagen Inc., Mansfield, MA, USA) to provide a supplementary nesting option in consideration of the substantial increase in cage size.

### *Alcohol drinking experiments*

For experiments examining effects of intranasal (IN) oxytocin (OXT) and intraperitoneal (IP) LIT-001 on alcohol (and water) consumption, adult female and male prairie voles (OXT, *n:* females vehicle=29, females 5mg/kg=13, females 10mg/kg=14, males vehicle=27, males 5mg/kg=12, males 10mg/kg=16; 75-177 days (d), of age at start of experiment; LIT-001, *n*: females vehicle=15, female LIT-001=16, males vehicle=12, males LIT-001=11; 76-138d) were given *ad libitum* access to alcohol in a continuous access two-bottle (alcohol, water) choice (CA-2BC) in HM-2 cages. Adult male and female prairie voles were implanted with RFID microchips (UNO MICRO ID/8, ISO Transponder, 2.12 x 8mm) under mild isoflurane anesthesia and allowed to recover in their home cages for 2d. Animals were then assigned with same-sex cagemates (3-4 animals/cage) to HM2 cages and allowed 2 d to habituate to the cage, with access to the channels blocked and water provided via 25 milliliter (ml) glass tubes equipped with sipper tops through the cage top. Following habituation, access to channels was opened - one bottle contained water and one bottle contained a 5% (v/v) ethanol in water solution (diluted from 95%, v/v, EtOH). Bottle position (front or back) was counterbalanced across cages and remained consistent throughout the course of the experiment. Access remained *ad libitum* in a CA-2BC procedure for 5d to allow for collection of baseline measures of consumption.

To habituate animals to intranasal IN administration or IP injection procedures, animals received vehicle treatment for 3d prior to treatment. In the OXT study, 25 microliters (µl) of saline was administered intranasally via a Hamilton syringe connected to cannula tubing and blunt cannula needle (33 gauge, 2.8 mm length; Plastics One, Roanoke, VA). In the LIT-001 study, vehicle was administered IP at a volume of 10 ml/kg of body weight. Animals were assigned to treatment or control groups pseudo-randomly to ensure relatively equal levels of baseline consumption and genetic diversity across groups.

On the treatment day, treatment animals received drug (OXT, IN or LIT-001, IP), while controls received vehicle only. A mixed-cage design - with treatment and control animals in the same cage - was employed. Treatments were administered at 08:30 on the treatment day in a between-subjects design. In the OXT experiments, two animals per cage received one of the doses of OXT while the other two animals in the cage received saline (vehicle control). In the LIT-001 experiment, two animals per cage received LIT-001, while the other two animals received 5% dimethyl sulfoxide in saline (vehicle control). If animals were housed at 3 per cage, at least one animal per cage received a different treatment than that of their cagemates, and the distribution of treatment was balanced across cohorts. Alcohol and water consumption were measured for a full 24hrs post-treatment (Figure S1). Doses were based on those previously used in investigations of OXT’s effects on central OXT systems in prairie voles [1, 2] and our previous study [3]; dose calculations were based on average animal weight. Treatment time was based on previous observation of slightly higher consumption of alcohol during the light versus the dark phase of circadian cycle [3-5] and occurrence of slightly higher drinking between 1-3 hrs after lights-on in pilot experiments. The dose of LIT-001 was based on previous demonstrations of restorative effects on social behaviors in genetically modified mice [6].

The distribution of many measures was not normal. Therefore, non-parametric statistics were used. Specifically, Kruskal-Wallis tests were used to examine differences between subgroups (control female, control male, 5 mg/kg OXT-treated female, 5 mg/kg OXT-treated male, 10 mg/kg OXT-treated female and 10 mg/kg OXT-treated male). When significant differences were found, significant pairwise comparisons were followed up with Mann-Whitney U tests to confirm the differences between specific groups.

*RT-PCR for RAGE mRNA*

Experimentally naïve adult female and male (*n* = 6, 3/sex, age approximately 90 days) prairie voles were euthanized via CO_2_ inhalation. RNA was isolated using TRIzol reagent (ThermoFisher, Waltham, MA, USA). The qRT-PCR reaction was performed using QuantiTect RT-PCR SYBR Green (Qiagen, Germantown, MD, USA) using an Mx3000p thermocycler (Stratagene, Bellingham, WA, USA) with primers 5’-AGAAGGTGGAACAGTCGCTC-3’ and 5’-CACCCACAAGAGCCTGTGATA-3’. The primers were designed based on the NCBI predicted transcript for Microtus ochrogaster advanced glycosylation end-product specific receptor (XM_005370772.3). Primers for the prairie vole oxytocin receptor and prairie vole house-keeping gene *Pgk1* (based on [7]) served as positive controls.

*Immunohistochemistry for RAGE protein*

Experimentally-naïve adult female and male voles (*n* = 10, 5/sex 75-179d) were euthanized via CO_2_ inhalation and perfused with 2% paraformaldehyde (PFA) in 0.1M phosphate-buffered saline (PBS). Brains were extracted and fixed in 2% PFA/PBS for 24 – 48hr, then cryoprotected in increasing concentrations of (20%, 30%) sucrose with 0.1% sodium azide in PBS for 24hr. Tissue was sliced in 40µm coronal sections and stored in (0.1%) sodium azide in PBS until the assay. Slices containing hypothalamic regions were selected for analysis and regions of interest were determined using the mouse brain atlas [8]. Areas of particular interest were based on previous demonstrations of RAGE expression in the human and mouse brain [9, 10]. The anti-RAGE antibody Abcam 3611 (1:1,000, Abcam, Waltham, MA) was used as the primary antibody, along with an anti-rabbit (produced in goat) secondary antibody (Vector Laboratories, Burlingame, CA) and signal was amplified using a VECTASTAIN^®^ ABC-HRP kit (Vector Laboratories, Burlingame, CA). Finally, tissue was stained using a metal enhanced diaminobenzidine substrate kit (ThermoFisher Scientific, Waltham, MA) and visualized using a Leica DM4000 bright-field microscope. Control tissue was processed identically with the exclusion of the primary anti-RAGE antibody and was assayed simultaneously.

### *Liquid chromatography-tandem triple quadrupole mass spectrometry*

Adult female and male prairie voles (75- 207d) were assigned to control (*n* =28; females = 14) or treatment (*n* = 29; females = 15) groups pseudo-randomly to ensure genetic diversity across groups. Whole brain tissue samples (~650 mg) were homogenized, subjected to protein precipitation and solid-phase extraction, and analyzed for concentrations (pg/mg) of exogenous oxytocin-(*leucine*-5,5,5-d_3_, *glycine*-2,2-d_2_) trifluoroacetate salt (d5 OXT) using a Shimadzu Nexera-LCMS-8060 (Shimadzu, Kyoto, Japan) liquid chromatograph-triple quadrupole mass spectrometer. This assay was a modification of an assay previously developed, validated, and utilized by the Endocrine Technologies Core (ETC) at Oregon National Primate Research Center (ONPRC) for analysis of OXT and d5 OXT in nonhuman primate samples [11].

In 5 (1 control female, 2 control males, 1 pre-treated female and 1 pre-treated male) out of 57 cases levels were above or below 2 standard deviations from the mean and thus removed from analysis. In cases when no d5 OXT was detected, the concentration was entered as zero (17 total).

The considerable number of zero values skewed the distribution and thus a non-parametric Kruskal-Wallis test was used to examine difference between subgroups (control IN female, control IN male, FPS ZM1 pre-treated IN female, FPS ZM1 pre-treated IN male, control IP female, control IP male, FPS ZM1 pre-treated IP female, FPS ZM1 pre-treated IP male). When significant differences were found, significant pairwise comparisons were followed up with Mann-Whitney U tests to confirm significant differences between groups.

**Supplemental Results**

# *RAGE expression in the prairie vole brain*

RAGE transcripts were successfully amplified from both male and female hypothalamic samples. Cycle threshold (Ct) levels for RAGE mRNA were similar to Ct levels of OXTR mRNA (~ 26-27). RAGE immunoreactivity was observed throughout the brain, including hypothalamus, areas surrounding the ventricles (Figure 3A), choroid plexus (Figure S5A) and the hippocampus (Figure S5C). Qualitative analysis showed similar expression patterns observed in females and males. No RAGE immunoreactivity was detected in control tissue in the absence of the primary anti-RAGE antibody (Figures 3B, S5B and S5D), confirming specificity of staining. These results demonstrate the presence and wide distribution of RAGE in the prairie vole brain, as well as confirm expression in brain areas where expression is also observed in humans and mice [9, 10].

**Supplemental Figures**

**Figure S1**


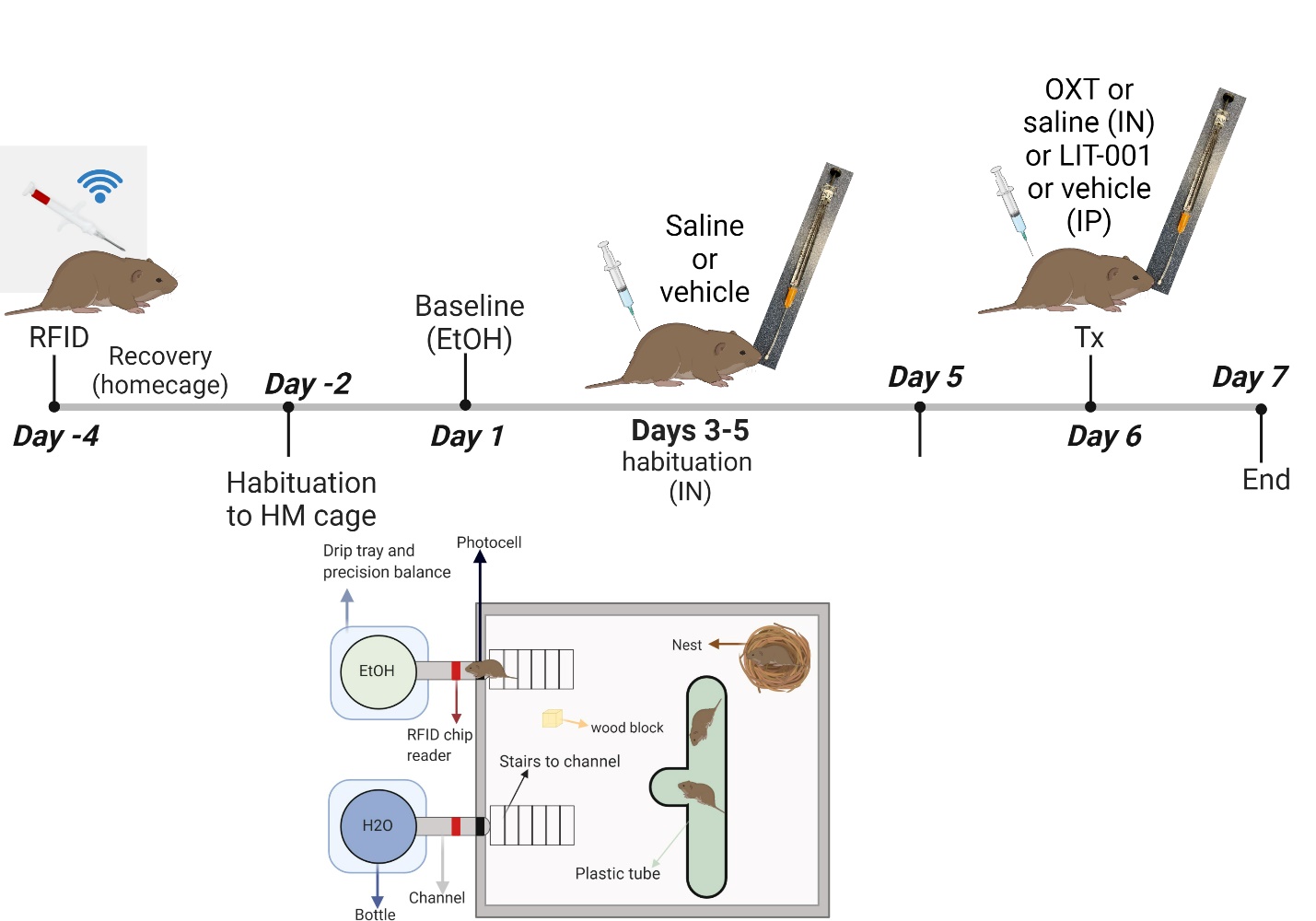


**Figure S1: Experimental timeline of alcohol drinking experiments.** As described above, animals were implanted with RFID microchips and allowed 2 days to recover in home cages before 2 days of habituation to HM2 cages. Five days of baseline drinking in a CA-2BC paradigm were used to pseudo-randomly assign animals to treatment (OXT or LIT-001) and control (vehicle) groups. To habituate animals to IN or IP procedures, vehicle was administered during the last 3 days of baseline. Consumption was measured for 24hrs post-treatment.

**Figure S2**

**
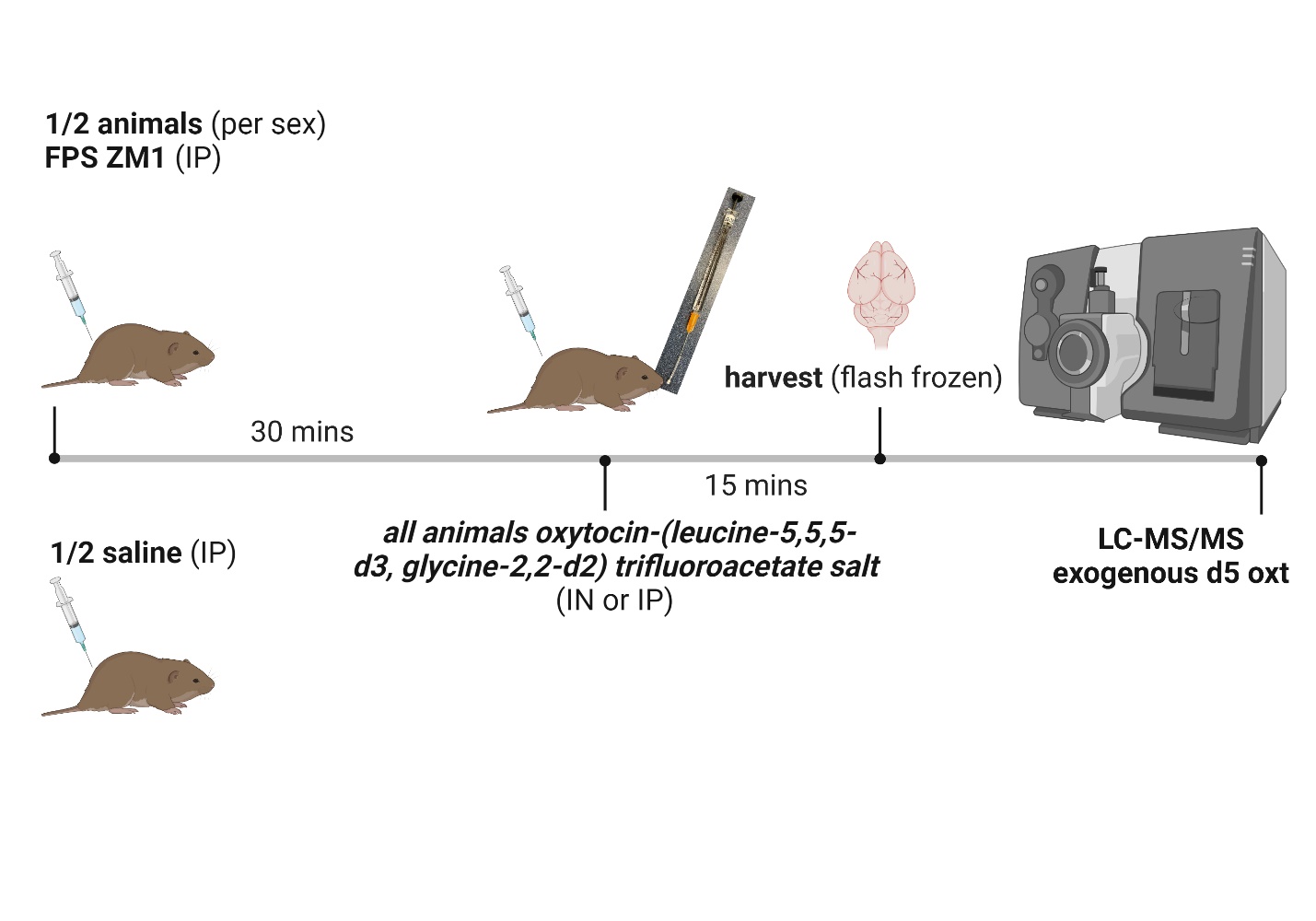
**

**Figure S2: Experimental timeline of RAGE antagonist and LC-MS/MS experiment.** As described above, half of the animals (per sex) were given the RAGE antagonist FPS ZM1 (IP) and half were given saline (IP, vehicle control) 30 mins prior to all animals receiving d5 OXT (IP or IN). After 15 mins, animals were anesthetized and perfused. Brains were harvested and flash frozen in isopentane. Whole brain samples were analyzed using LC-MS/MS.

**Figure S3**

**
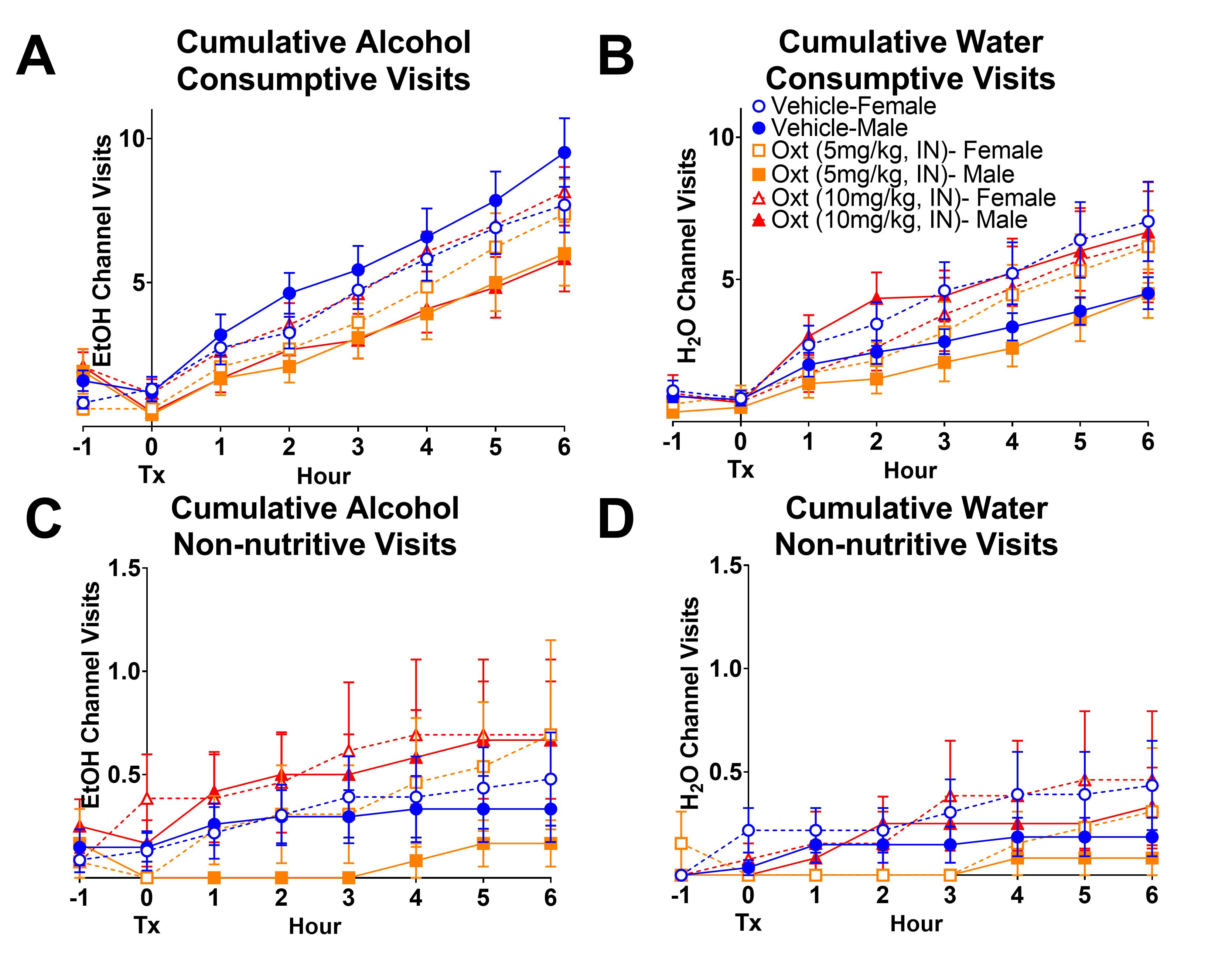
**

**Figure S3: Cumulative measures of visits to drinking channels from 1 hr pre- through 6hrs post- intranasal oxytocin treatment.** No significant differences in alcohol (A) or water (B) consumptive visits or in alcohol (C) or water (D) non-nutritive visits were observed at any time point. Data are presented as mean ± standard error of mean. Tx denotes time of treatment. Note: -1hr time point is included to demonstrate lack of differences between groups prior to treatment and is defined as the cumulative measure during the 1hr prior to treatment. Cumulative measures post-treatment begin at the 0hr time point which encompasses the time of treatment (Tx) through the first hour. *N:* female vehicle = 29, female 5mg/kg OXT =13, female 10mg/kg OXT = 14, male vehicle = 27, male 5mg/kg OXT = 12, male 10mg/kg OXT = 16.

**Figure S4**


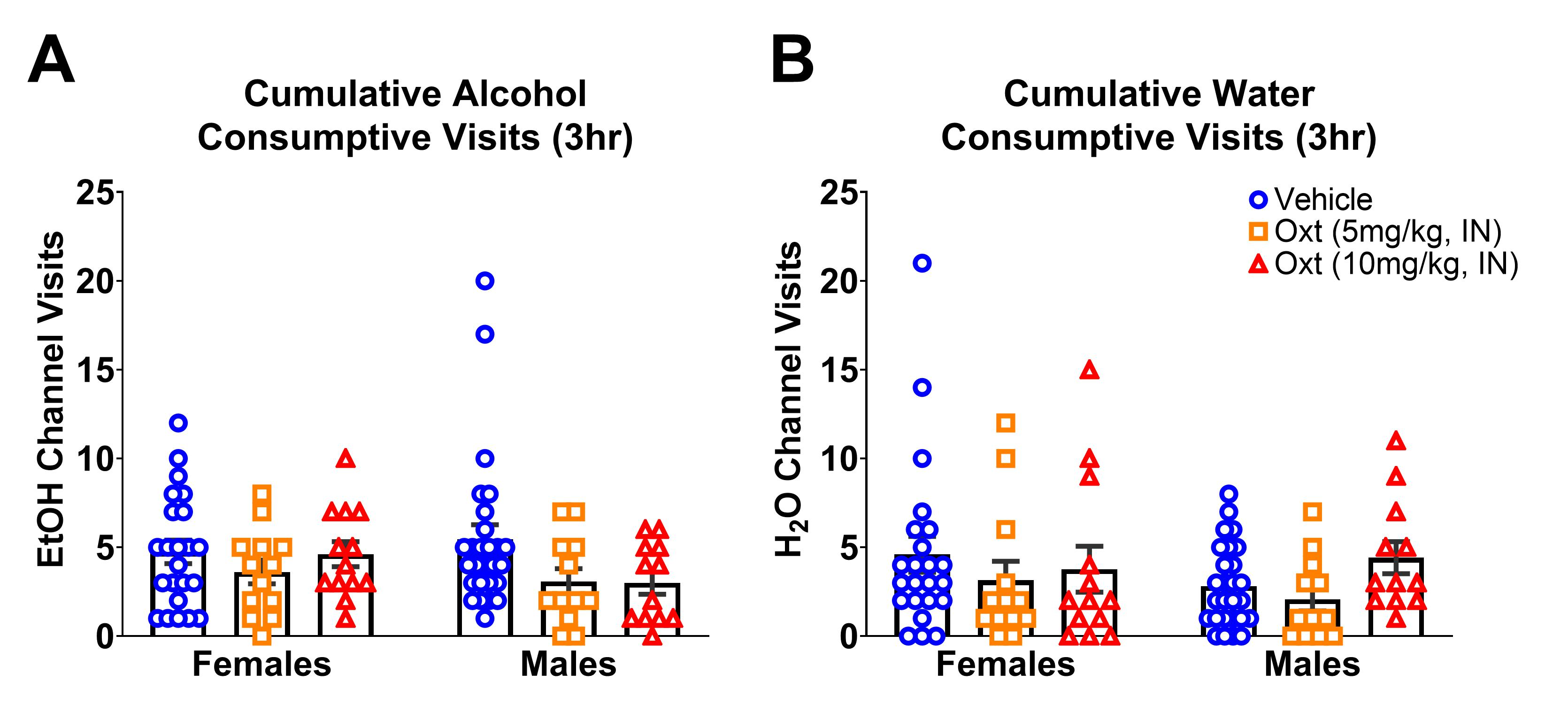


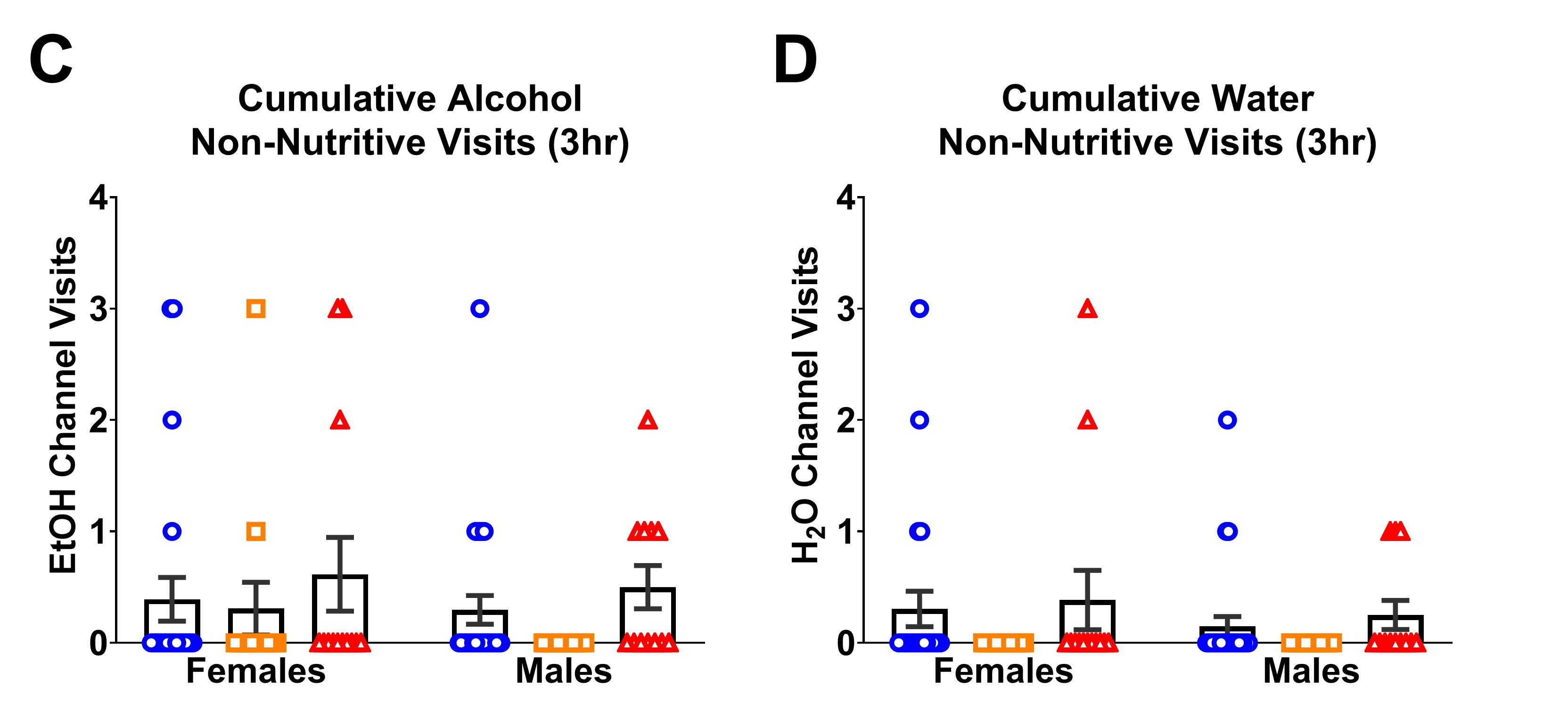


**Figure S4: Cumulative measures of visits to drinking channels 3hr following intranasal oxytocin treatment.** No statistically significant effects were observed on either alcohol (A) or water (B) consumptive visits or alcohol (C) or water (D) non-nutritive visits. Data are presented as mean ± standard error of mean. N values are the same as in Figure S3.

**Figure S5**


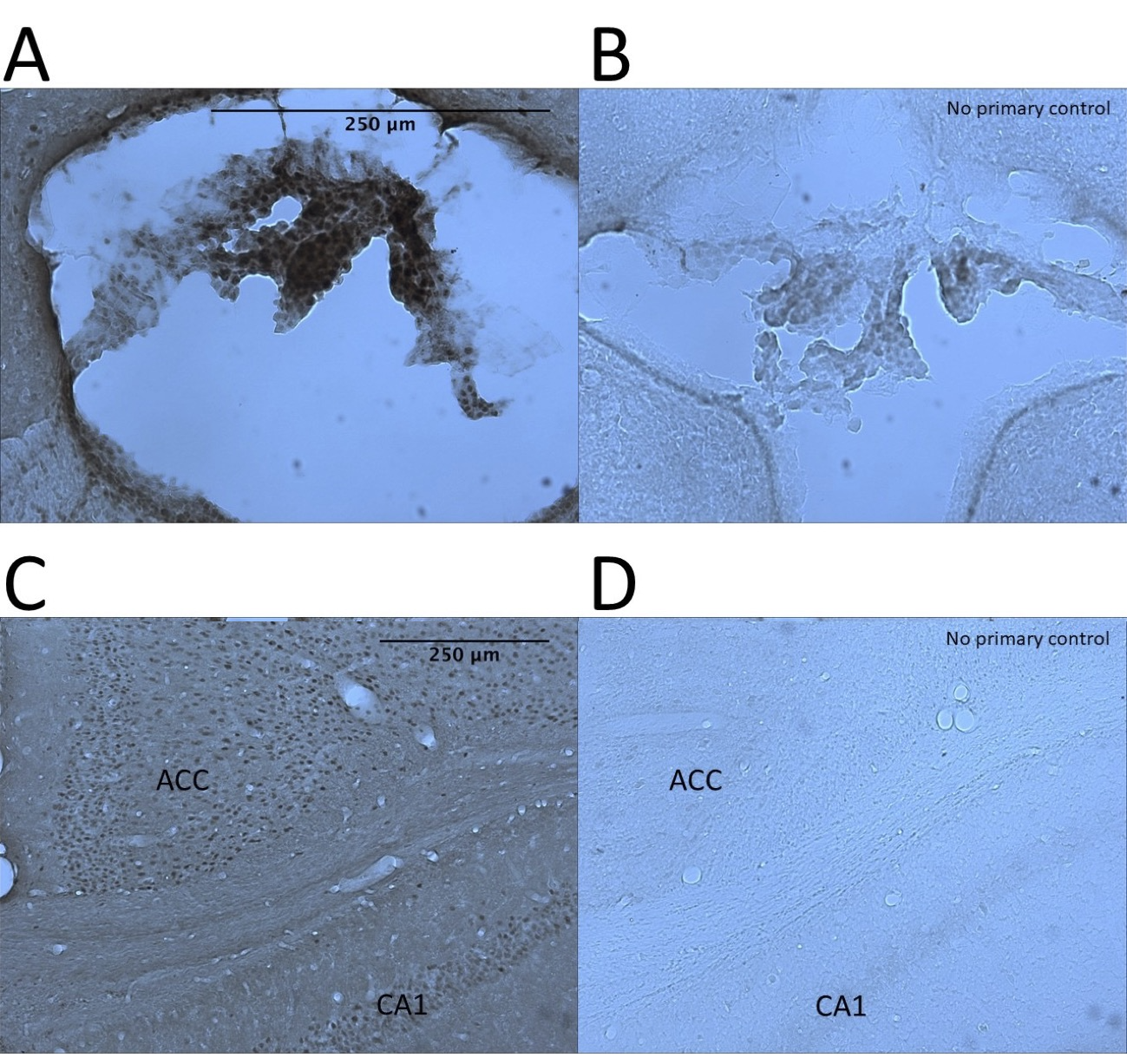


**Figure S5: Representative photomicrographs of RAGE-immunoreactivity in the prairie vole brain.** RAGE immunoreactivity was observed widely in the prairie vole brain, including areas surrounding the ventricles, the choroid plexus (A) and the hippocampus (C). Staining was confirmed via comparison to no-primary controls (B and D). Images A and B are at 20X objective magnification, images C and D are at 10X objective magnification. ACC = anterior cingulate cortex; CA1 = CA1 region of the hippocampus.

**Figure S6**


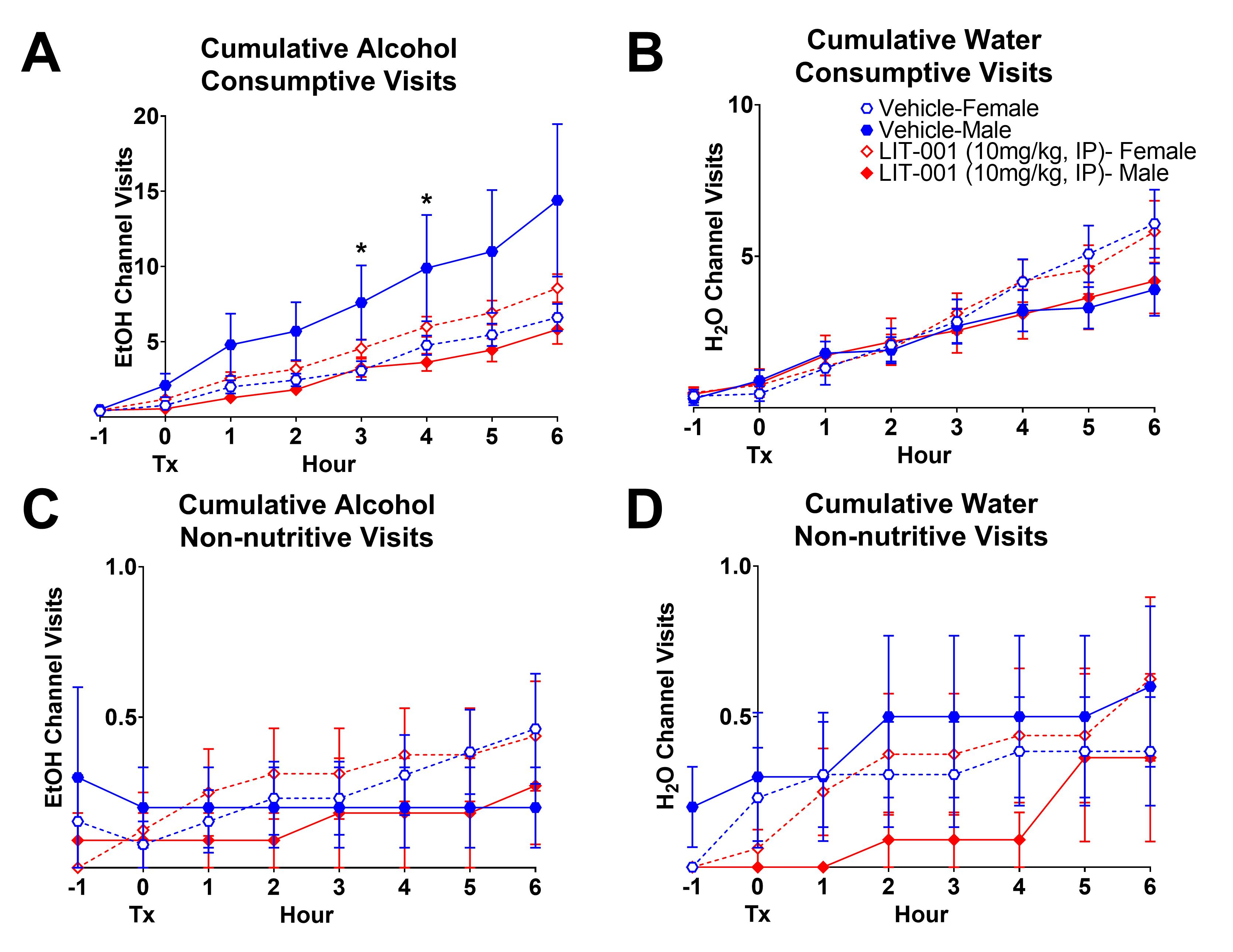


**Figure S6: Cumulative measures of visits to drinking channels from 1 hr pre- through 6hrs post- LIT-001 treatment.** Significant differences in alcohol consumptive visits were noted at 3- and 4hrs (A), but no differences in water consumptive visits (B) or alcohol (C) or water (D) non-nutritive visits were found. Data are presented as mean ± standard error of mean. **p* < 0.05, Kruskal-Wallis test. Tx denotes time of treatment. Note: -1hr time point included to demonstrate lack of differences between groups prior to treatment. Cumulative measures post-treatment begin at the 0 hr time point which encompasses the time of treatment (Tx) through the first hour. *N*: female vehicle = 15, female LIT-001 = 16, male vehicle = 12, male LIT-001 = 11.

**Figure S7**


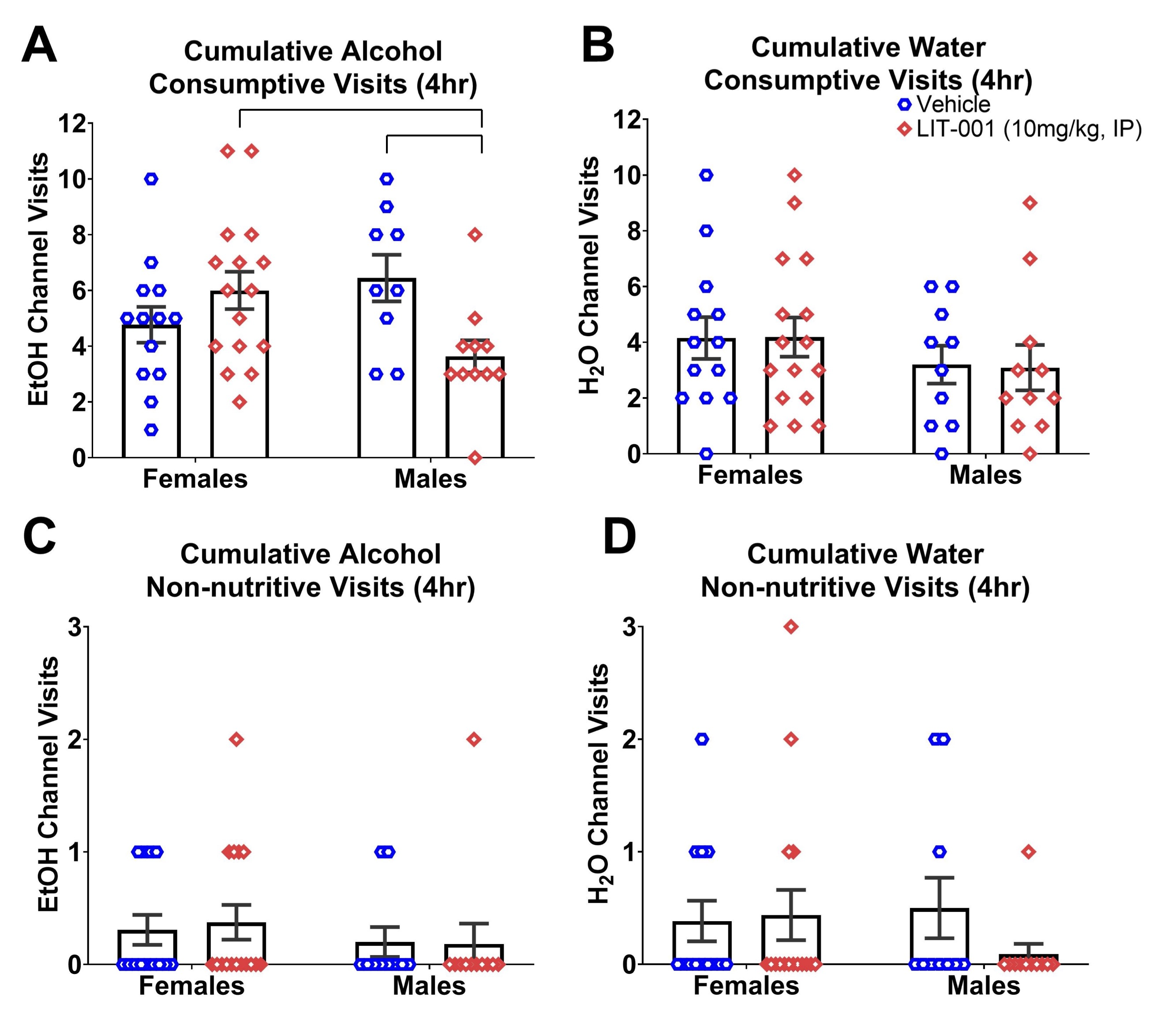


**Figure S7: Cumulative measures of visits to drinking channels 4hr following LIT-001 treatment.** LIT-001 treated males were made fewer consumptive visits (CVs) to the alcohol channel (A) than control males and LIT-001-treated females, without differences in water cumulative visits (B), or alcohol (C) or water (D) non-nutritive visits. Data are presented as mean ± standard error of mean (SEM). Brackets indicate statistically significant differences between groups at *p*s < 0.05, Mann-Whitney test. N values are the same as in Figure S6.

**Supplemental References:**

1. Bales, K., et al., *Chronic intranasal oxytocin causes long-term impairments in partner preference formation in male prairie voles.* Biological Psychology, 2013. **74**: p. 180-8.

2. Guoynes, C., et al., *Chronic Intranasal Oxytocin has Dose-dependent Effects on Central Oxytocin and Vasopressin Systems in Prairie Voles (Microtus ochrogaster). - PubMed - NCBI.* Neuroscience, 2018. **369**: p. 292-302.

3. Walcott, A.T. and A.E. Ryabinin, *Assessing effects of oxytocin on alcohol consumption in socially housed prairie voles using radio frequency tracking.* Addict Biol, 2020: p. e12893.

4. Anacker, A.M., et al., *Prairie voles as a novel model of socially facilitated excessive drinking.* Addict Biol, 2011. **16**(1): p. 92-107.

5. Anacker, A.M.J. and A.E. Ryabinin, *Identification of subpopulations of prairie voles differentially susceptible to peer influence to decrease high alcohol intake.* Front Pharmacol, 2013. **4**.

6. Frantz, M.C., et al., *LIT-001, the First Nonpeptide Oxytocin Receptor Agonist that Improves Social Interaction in a Mouse Model of Autism.* J Med Chem, 2018. **61**(19): p. 8670-8692.

7. Perkeybile, A., et al., *Early nurture epigenetically tunes the oxytocin receptor.* Psychoneuroendocrinology, 2019. **99**.

8. Franklin, K.B. and G. Paxinos, *The Mouse Brain in Stereotaxic Coordinates.* Academic Press, 2008(3).

9. Cheng, C., et al., *Expression profiling of endogenous secretory receptor for advanced glycation end products in human organs.* Mod Pathol, 2005. **18**(10): p. 1385-96.

10. Harashima, A., et al., *Identification of mouse orthologue of endogenous secretory receptor for advanced glycation end-products: structure, function and expression.* Biochem J, 2006. **396**(1): p. 109-15.

11. Lee, M.R., et al., *Labeled oxytocin administered via the intranasal route reaches the brain in rhesus macaques.* Nat Commun, 2020. **11**(1): p. 2783.
