# Supplementary material for: Male-selective effects of oxytocin agonism on alcohol intake: behavioral assessment in socially housed prairie voles and involvement of RAGE
Source: Neuropsychopharmacology. 2022 Nov 11;48(6):920–8. doi: 10.1038/s41386-022-01490-3 (PMC10156683; doi:10.1038/s41386-022-01490-3)
Supplement: Supplementary file 2 — Supplemental Table [file 41386_2022_1490_MOESM2_ESM.docx]

| **Experiment** | **Figure and Panel** | **Dependent Variable** | **Statistical Test Used** | **Statistical Report** |
| --- | --- | --- | --- | --- |
| 1 | 1A | EtOH intake 1hr post-tx | Kruskal-Wallis test, Factor: Treatment group | *H*(5) = 9.92, *p* = 0.08 |
| 1 | 1A | EtOH intake 2hr post-tx | Kruskal-Wallis test, Factor: Treatment group | *H*(5) = 13.19, *p* = 0.02 |
| 1 | 1B | H2O intake 2hr post-tx | Kruskal-Wallis test, Factor: Treatment group | *H*(5) = 11.78, *p* = 0.04 |
| 1 | 1A | EtOH intake 3hr post-tx | Kruskal-Wallis test, Factor: Treatment group | *H*(5) = 14.03, *p* = 0.02 |
| 1 | 1B | H2O intake 3hr post-tx | Kruskal-Wallis test, Factor: Treatment group | *H*(5) = 9.04, *p* = 0.11 |
| 1 | 1C | EtOH drink size 2hr post-tx | Kruskal-Wallis test, Factor: Treatment group | *H*(5) = 13.06, *p* = 0.02 |
| 1 | 1D | H2O drink size 2hr post-tx | Kruskal-Wallis test, Factor: Treatment group | *H*(5) = 12.34, *p* = 0.03 |
| 1 | 1C | EtOH drink size 3hr post-tx | Kruskal-Wallis test, Factor: Treatment group | *H*(5) = 11.46, *p* = 0.04 |
| 1 | 1C | EtOH drink size 4hr post-tx | Kruskal-Wallis test, Factor: Treatment group | *H*(5) = 10.86, *p* = 0.054 |
| 1 | 1C | EtOH drink size 5hr post-tx | Kruskal-Wallis test, Factor: Treatment group | *H*(5) = 12.55, *p* = 0.03 |
| 1 | 2A | EtOH intake 3hrs post-tx | Mann Whitney U test, comparison: 5mg/kg treated males vs. saline treated male controls | 5mg/kg OXT males: *M* = 0.72, ±*SEM* = 0.16; saline males: *M* = 1.54, ±*SEM* = 0.17  *U*(*N*_control male_ = 27, *N*_5 mg/kg OXT male_ = 12) = 73.00, *z* = -2.71, *p* = 0.01, Cohen’s d=1.06 |

**Supplementary Table 1**

| **Experiment** | **Figure and Panel** | **Dependent Variable** | **Statistical Test Used** | **Statistical Report** |
| --- | --- | --- | --- | --- |
| 1 | 2A | EtOH intake 3hrs post-tx | Mann Whitney U test, comparison: 10mg/kg treated males vs. saline treated male controls | 10mg/kg OXT males: *M* = 0.82, ±*SEM* = 0.19; saline males:  *M* = 1.54, ±*SEM* = 0.17  *U*(N_control male_ = 27, *N*_10 mg/kg OXT male_ = 12) = 86.00, *z* = -2.31, *p* = 0.02, Cohen’s d=0.90 |
| 1 | 2C | EtOH drink size 3hrs post-tx | Mann Whitney U test, comparison: 5mg/kg treated males vs. saline treated male controls | 5mg/kg OXT males: *M* = 0.85, ±*SEM* = 0.20; saline males:  *M* = 1.98, ±*SEM* = 0.24  *U*(*N*_control male_ = 27, *N*_5 mg/kg OXT male_ = 12) = 71.50, *z* = -2.75, *p* = 0.01, Cohen’s d=1.05 |
| 1 | 2C | EtOH drink size 3hrs post-tx | Mann Whitney U test, comparison: 10mg/kg treated males vs. saline treated male controls | 10mg/kg OXT males: *M* = 1.03, ±*SEM* = 0.28; saline males:  *M* = 1.98, ±*SEM* = 0.24  *U*(*N*_control male_ = 27, *N*_10 mg/kg OXT male_ = 12) = 85.50, *z* = -2.33, *p* = 0.02, Cohen’s d=0.83 |
| 1 | 2A | EtOH intake 3hrs post-tx | Mann Whitney U test, comparison: 5mg/kg treated males vs. 5mg/kg treated females | 5mg/kg OXT males: *M* = 0.72, ±*SEM* = 0.16; 5mg/kg OXT females: *M* = 1.44, ±*SEM* = 0.26  *U*(*N*_5 mg/kg OXT female_ = 13, *N*_5 mg/kg OXT male_ = 12) = 40.00, *z* = -2.07, *p* = 0.04, Cohen’s d=0.96 |
| 1 | 2A | EtOH intake 3hrs post-tx | Mann Whitney U test, comparison: 5mg/kg treated males vs. 10mg/kg treated females | 5mg/kg OXT males: *M* = 0.72, ±*SEM* = 0.16; 10mg/kg OXT females: *M* = 1.64, ±*SEM* = 0.20  *U*(*N*_10 mg/kg OXT female_ = 13, *N*_5 mg/kg OXT male_ = 12) = 24.00, *z* = -2.94, *p* = 0.01, Cohen’s d=1.51 |
| 1 | 2C | EtOH drink size 3hrs post-tx | Mann Whitney U test, comparison: 5mg/kg treated males vs. 5mg/kg treated females | 5mg/kg OXT males: *M* = 0.85, ±*SEM* = 0.20; 5mg/kg OXT females: *M* = 1.53, ±*SEM* = 0.29  *U*(*N*_5 mg/kg OXT female_ = 13, *N*_5 mg/kg OXT male_ = 12) = 48.50, *z* = -1.61, *p* = 0.11, Cohen’s d=0.79 |
| 1 | 2C | EtOH drink size 3hrs post-tx | Mann Whitney U test, comparison: 5mg/kg treated males vs. 10mg/kg treated females | 5mg/kg OXT males: *M* = 0.85, ±*SEM* = 0.20; 10mg/kg OXT females: *M* = 1.63, ±*SEM* = 0.17  *U*(*N*_10 mg/kg OXT female_ = 13, *N*_5 mg/kg OXT male_ = 12) = 33.00, *z* = -2.45, *p* = 0.01, Cohen’s d=1.26 |
| 1 | 2A | EtOH intake 3hrs post-tx | Mann Whitney U test, comparison: 10mg/kg treated males vs. 10mg/kg treated females | 10mg/kg OXT males: *M* = 0.82, ±*SEM* = 0.19; 10mg/kg OXT females: *M* = 1.64, ±*SEM* = 0.20  *U*(*N*_10 mg/kg OXT female_ = 13, *N*_10 mg/kg OXT male_ = 12) = 27.00, *z* = -2.77, *p* = 0.01, Cohen’s d=1.24 |
| 1 | 2C | EtOH drink size 3hrs post-tx | Mann Whitney U test, comparison: 10mg/kg treated males vs. 10mg/kg treated females | 10mg/kg OXT males: *M* = 1.03, ±*SEM* = 0.28; 10mg/kg OXT females: *M* = 1.63, ±*SEM* = 0.17  *U*(*N*_10 mg/kg OXT female_ = 13, *N*_10 mg/kg OXT male_ = 12) = 43.50, *z* = -1.88, *p* = 0.06, Cohen’s d=0.78 |
| 2 | 3 | d5 OXT (pg/mg) | Kruskal-Wallis test, Factor: Treatment group | *H*(7) = 18.38, *p* = 0.01 |
| 2 | 3 | d5 OXT (pg/mg) | Mann Whitney U test, comparison: FPS ZM1 pre-treated IN d5 OXT males vs. saline pre-treated IN d5 OXT male controls | Antagonist IN males: *M* = 0.00, ±*SEM* = 0.00; control IN males:  *M* = 0.01, ±*SEM* = 0.002  *U*(*N*_antagonist male_ = 6, *N*_control male_ = 6) = 3.00, *z* = -2.68, *p* = 0.02, Cohen’s d=3.13 |
| 2 | 3 | d5 OXT (pg/mg) | Mann Whitney U test, comparison: FPS ZM1 pre-treated IN d5 OXT females vs. FPS ZM1 pre-treated IP d5 OXT females | Antagonist IN females: *M* = 0.006, ±*SEM* = 0.003; Antagonist IP females:  *M* = 0.05, ±*SEM* = 0.02  *U*(*N*_antagonist IN female_ = 7, *N*_antagonist IP female_ = 7) = 8.50, *z* = -2.07, *p* = 0.04, Cohen’s d=1.26 |
| 2 | 3 | d5 OXT (pg/mg) | Mann Whitney U test, comparison: FPS ZM1 pre-treated IN d5 OXT females vs. FPS ZM1 pre-treated IP d5 OXT males | Antagonist IN females:  *M* = 0.006, ±*SEM* = 0.003; Antagonist IP males:  *M* = 0.05, ±*SEM* = 0.01  *U*(*N*_antagonist IN female_ = 7, *N*_antagonist IP male_ = 7) = 6.00, *z* = -2.39, *p* = 0.02, Cohen’s d=2.44 |
| 2 | 3 | d5 OXT (pg/mg) | Mann Whitney U test, comparison: FPS ZM1 pre-treated IN d5 OXT males vs. FPS ZM1 pre-treated IP d5 OXT males | Antagonist IN males: *M* = 0.00, ±*SEM* = 0.00; Antagonist IP males: *M* = 0.05, ±*SEM* = 0.01  *U*(*N*_antagonist IN male_ = 6, *N*_antagonist IP male_ = 7) = 0.00, *z* = -3.16, *p* = 0.002, Cohen’s d=2.94 |
| 2 | 3 | d5 OXT (pg/mg) | Mann Whitney U test, comparison: FPS ZM1 pre-treated IN d5 OXT males vs. FPS ZM1 pre-treated IP d5 OXT females | Antagonist IN males:  *M* = 0.00, ±*SEM* = 0.00; Antagonist IP females: *M* = 0.05, ±*SEM* = 0.02  *U*(*N*_antagonist IN male_ = 7, *N*_antagonist IP female_ = 7) = 0.00, *z* = -3.16, *p* = 0.002, Cohen’s d=1.43 |
| 3 | 4A | EtOH intake 4hr post-tx | Kruskal-Wallis test, Factor: Treatment group | *H*(3) = 7.92, *p* = 0.05 |
| 3 | 4B | H2O intake 4hr post-tx | Kruskal-Wallis test, Factor: Treatment group | *H*(3) = 2.58, *p* = 0.46 |
| 3 | 4C | EtOH drink size 4hr post-tx | Kruskal-Wallis test, Factor: Treatment group | *H*(3) = 5.52, *p* = 0.14 |
| 3 | 4D | H2O drink size 4hr post-tx | Kruskal-Wallis test, Factor: Treatment group | *H*(3) = 1.20, *p* = 0.75 |
| 3 | 4E | EtOH CV 4hr post-tx | Kruskal-Wallis test, Factor: Treatment group | *H*(3) = 9.35, *p* = 0.03 |
| 3 | 4F | H2O CV 4hr post-tx | Kruskal-Wallis test, Factor: Treatment group | *H*(3) = 2.08, *p* = 0.56 |
| 3 | 4G | EtOH NNV 4hr post-tx | Kruskal-Wallis test, Factor: Treatment group | *H*(3) = 1.82, *p* = 0.61 |
| 3 | 4H | H2O NNV 4hr post-tx | Kruskal-Wallis test, Factor: Treatment group | *H*(3) = 1.97, *p* = 0.58 |
| 3 | 5A | EtOH intake 4hr post-tx | Mann Whitney U test, comparison: LIT-001 treated males vs. vehicle male controls | LIT-001 males: *M* = 1.36, ±*SEM* = 0.24; vehicle males: *M* = 2.66, ±*SEM* = 0.54  *U*(*N*_control male_ = 10, *N*_10 mg/kg LIT-001 male_ = 11) = 31.00, *z* = -1.69, *p* = 0.10, Cohen’s d=1.04 |
| 3 | 5A | EtOH intake 4hr post-tx | Mann Whitney U test, comparison: LIT-001 treated males vs. vehicle female controls | LIT-001 males: *M* = 1.36, ±*SEM* = 0.24; vehicle females: *M* = 2.71, ±*SEM* = 0.33  *U*(*N*_control female_ = 13, *N*_10 mg/kg LIT-001 male_ = 11) = 26.00, *z* = -2.64, *p* = 0.01, Cohen’s d=1.38 |
| 3 | 5A | EtOH intake 4hr post-tx | Mann Whitney U test, comparison: LIT-001 treated males vs. LIT-001 treated females | LIT-001 males: *M* = 1.36, ±*SEM* = 0.24; LIT-001 females:  *M* = 2.35, ±*SEM* = 0.24  *U*(*N*_10 mg/kg LIT-001 female_ = 16, *N*_10 mg/kg LIT-001 male_ = 11) = 40.00, *z* = -2.37, *p* = 0.02, Cohen’s d=1.14 |
| 3 | 5E | EtOH CV 4hr post-tx | LIT-001 treated males vs. vehicle male controls | LIT-001 males: *M* = 3.64, ±*SEM* = 0.58; vehicle males: *M* = 9.90, ±*SEM* = 3.54  *U*(*N*_control male_ = 10, *N*_10 mg/kg LIT-001 male_ = 11) = 19.50, *z* = -2.56, *p* = 0.01, Cohen’s d=0.84 |
| 3 | 5E | EtOH CV 4hr post-tx | Mann Whitney U test, comparison: LIT-001 treated males vs. LIT-001 treated females | LIT-001 males: *M* = 3.64, ±*SEM* = 0.58; LIT-001 females: *M* = 6.00, ±*SEM* = 0.67  *U*(*N*_10 mg/kg LIT-001 female_ = 16, *N*_10 mg/kg LIT-001 male_ = 11) = 43.00, *z* = -2.26, *p* = 0.03, Cohen’s d=1.02 |
